# Predictive SIRT dosimetry based on a territorial model

**DOI:** 10.1186/s40658-017-0192-5

**Published:** 2017-10-31

**Authors:** Nadine Spahr, Philipp Schilling, Smita Thoduka, Nasreddin Abolmaali, Andrea Schenk

**Affiliations:** 1 Fraunhofer Institute for Medical Image Computing MEVIS, Lübeck, Maria-Goeppert-Straße 3, Lübeck, 23562 Germany; 2 Ressort TuW, Abt. MIT, FB Strahlenphysik, Städtisches Klinikum Dresden-Friedrichstadt, Friedrichstraße 41, Dresden, 01067 Germany; 3 Department of Radiology, Städtisches Klinikum Dresden-Friedrichstadt, Friedrichstraße 41, Dresden, 01067 Germany; 4 Fraunhofer Institute for Medical Image Computing MEVIS, Am Fallturm 1, Bremen, 28359 Bremen Germany

**Keywords:** Yttrium-90 microspheres, Radioembolization, SIRT, Dosimetry

## Abstract

**Background:**

In the planning of selective internal radiation therapy (SIRT) for liver cancer treatment, one major aspect is to determine the prescribed activity and to estimate the resulting absorbed dose inside normal liver and tumor tissue. An optimized partition model for SIRT dosimetry based on arterial liver territories is proposed. This model is dedicated to characterize the variability of dose within the whole liver. For an arbitrary partition, the generalized absorbed dose is derived from the classical partition model. This enables to consider normal liver partitions for each arterial perfusion supply area and one partition for each tumor for activity and dose calculation. The proposed method excludes a margin of 11 mm emitting range around tumor volumes from normal liver to investigate the impact on activity calculation. Activity and dose calculation was performed for five patients using the body-surface-area (BSA) method, the classical and territorial partition model.

**Results:**

The territorial model reaches smaller normal liver doses and significant higher tumor doses compared to the classical partition model. The exclusion of a small region around tumors has a significant impact on mean liver dose. Determined tumor activities for the proposed method are higher in all patients when limited by normal liver dose. Activity calculation based on BSA achieves in all cases the lowest amount.

**Conclusions:**

The territorial model provides a more local and patient-individual dose distribution in normal liver taking into account arterial supply areas. This proposed arterial liver territory-based partition model may be used for SPECT-independent activity calculation and dose prediction under the condition of an artery-based simulation for particle distribution.

## Background

Selective internal radiation therapy (SIRT) is a kind of brachytherapy used in interventional radiology to treat cancer [1]. In particular, patients with unresectable cancers, such as hepatocellular carcinoma, resistant to conventional chemotherapy and poorly accessible in conventional radiotherapy [2], or liver metastases, are eligible for SIRT. This type of therapy exploits the fact that the vascular system of tumor tissue differs from that of normal liver. Nearly 70–80% of a normal liver is supplied by the portal vein; the remainder is supplied by the hepatic artery [3]. However, liver tumors are usually supplied by arterial vessels. SIRT takes advantage of these supply characteristics for selective tumor treatment by transarterial radioembolization. During an interventional treatment session, yttrium-90 (Y-90)-labelled microspheres are ideally administered through a catheter into the tumor-feeding arteries. In practice, these arteries supply the tumors but also normal liver parenchyma. The microspheres embolize the capillaries and emit beta radiation due to their Y-90 radionuclide, thereby irradiating the embolized tumor and potential liver parenchyma regions.

Prior to SIRT, an evaluation procedure is performed for treatment planning purposes. The evaluation is necessary, and diagnostic angiography is used to select the best catheter positions for tumoral targeting and to identify digestive arteries arising from the hepatic artery. The latter are coiled during the evaluation procedure to avoid digestive damage. When the optimal catheter positions are identified, a radiopharmaceutical, e.g., technetium-99m (Tc-99m) macroaggregated albumin (MAA), is delivered to the hepatic artery. This step is an approach to simulate the treatment intervention using Tc-99m MAA as a surrogate for Y-90 microspheres. Afterwards, the MAA particle distribution is visualized by planar bremsstrahlung imaging or by SPECT imaging depending on the center and radiologist. The acquired MAA SPECT is then routinely used to determine the lung shunt fraction. It is also used in research studies for Y-90 activity and dose planning purposes. Empirical activity planning by the body-surface-area (BSA) method is inferior to the more advanced partition model (PM) [4], which additionally allows to predict mean dose values inside predefined organ partitions [5].

The PM introduces three main tissue compartments for SIRT dosimetry: lung, normal liver, and tumor. Based on the MAA SPECT/CT, the amount of deposited activity is determined for each partition. The absorbed dose can then be calculated. This model enables straightforward dose calculation in short computation times. However, assumptions and simplifications are made affecting the determined absorbed dose. The PM gathers large volumes of interest in one partition and assumes a uniform distribution of activity throughout the partition. Especially for normal liver, this might be critical in case of dose calculation. The PM would gather the whole normal liver activity and distribute it evenly over the whole normal liver partition during dose calculation. Regional activity distribution is not considered and also the handling of multiple tumors is not feasible.

Kao et al. [6] suggested an artery-specific partition modeling for radioembolization. Arterial regional margins are delineated for each catheter position via invasive catheter-directed CT hepatic angiography during the MAA intervention. Activity and dose planning according to the PM are performed for each region individually. The method of Kao et al. [6] does not take into account all patient-individual arterial territories at the same time but relies on the MAA SPECT/CT distribution, which is a composition of the whole administered activity. Activity assignment to individual catheter positions might not be feasible.

We propose an optimized, extended partition model, called territorial model (TM), introducing more partitions to the normal liver based on arterial liver territories [7]. In addition, we permit several tumor partitions for non-connected tumors or metastasis.

## Methods

In the following, we introduce the arterial liver territories that were used for the optimized partition model. Afterwards, the numerical details of SIRT dose calculation based on the partition model is described, the extended model is introduced, and activity calculation is presented briefly. Information about patient images and data analysis is given. Here, we focus on Y-90 resin SIR-Spheres^®;^ (Sirtex Medical Limited, North Sydney, Australia), but the approach is not limited to them.

### Determination of arterial liver territories

**Fig. 1:**
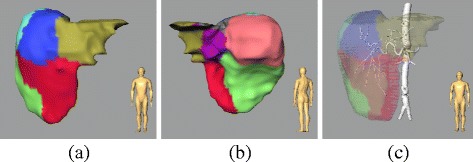
Visualization of 3D rendered liver territories from anterior (**a**) and posterior (**b**) view and translucent territories with opaque visualization of the arterial vessel system (**c**)

Arterial liver territories describe the supply area of arterial vessels based on the individual patient anatomy. An individual arterial vessel segmentation in CT data and a liver segmentation is used to determine arterial territories. The arterial vessel segmentation, typically represented as a set of voxels, is skeletonized, resulting in an abstract graph with ramifications as vertices and connecting edges. Each liver voxel is then assigned to one arterial branch by a model-based approach considering the shortest distance. The partitions are assigned based on the Couinaud segments. The applied method to determine arterial territories from an input vessel segmentation is given in detail in [7], where it was also evaluated for portal vein territories but not for arterial territories. An example of the calculated liver territories is given in Fig. 1. The accuracy depends on image resolution, image noise, and timing of contrast agent application. We additionally introduce a margin of 11 mm around tumors that will not be included into normal liver territories for activity calculation.

### SIRT dose calculation

The Medical Internal Radiation Dose (MIRD) Committee of the Society of Nuclear Medicine developed the PM approach for Y-90 microsphere dosimetry which is also recommended by the American Association of Physicists in Medicine (AAPM) since 2011 [3]. In general, it describes the interaction of the deposited radioactive seed with tissue. The mean absorbed dose *D*, or in short dose, specifies the amount of energy *Δ* *E* deposited in a mass of tissue *Δ* *m* from ionizing radiation. Formally, this can be described by

1 $$ D = \frac{\Delta E}{\Delta m},   $$

according to the ICRU (International Commission on Radiation Units and Measurements). In case of Y-90 radioembolization with numerous radioactive seeds, the absorbed dose *D* is under the assumption of local energy deposition more precisely given by

2 $$ D = \frac{\langle{E}\rangle A_{0} }{m} \int_{0}^{\infty} e^{-\ln(2) t/T_{1/2} } \mathrm{d} t = \frac{\langle{E}\rangle A_{0} }{m} \frac{T_{1/2}}{\ln(2)},   $$

with *A*_0_ being the activity in the tissue of interest, *m* the mass of the tissue, and 〈*E*〉 the average energy emitted per nuclear transition. The dose rate is calculated by integration over time *t* yielding the absorbed dose. *T*_1/2_ is the half-life of the radioactive source. With 〈*E*〉 equaling 0.9267 MeV [3] and a half-life of 64.04 h, we see that the constant term is

3 $$ \langle{E}\rangle \frac{T_{1/2}}{\ln(2)} = \frac{0.9267\left[\text{MeV}\right]\cdot 64.04\left[\mathrm{h}\right]}{\ln(2)} =49.38 \frac{\mathrm{Gy kg}}{\text{GBq}}.   $$

The absorbed dose can be expressed shortly by

4 $$ D[\text{Gy}] = 49.38 \left[\frac{\mathrm{Gy kg}}{\text{GBq}}\right] \frac{A_{0}\left[\text{GBq}\right] }{m\left[\text{kg}\right]}.   $$

#### Partition model

For a tissue of interest, a so-called partition, the mean absorbed dose can be determined using the PM. This model defines three partitions, lung, normal liver, and tumor, and enables calculation of absorbed doses for these partitions. One assumption is that the total delivered activity *A*_0_ meets the following requirement

5 $$ A_{0} = A_{\mathrm{L}} + A_{\text{NL}} + A_{\mathrm{T}},   $$

where *A*_L_ represents the activity shunted to the lung parenchyma. It is the abbreviation for *A*_Lung_ due to better readability. The same applies for *A*_NL_ instead of *A*_Normal liver_ and *A*_T_ instead of *A*_Tumor_. Here, the fraction of activity shunting into the lung, the so-called lung shunt fraction *L*, is determined from the MAA SPECT/CT image by the ratio of lung counts *c*_L_ to the sum of lung and liver counts

6 $$ L = \frac{c_{\mathrm{L}} }{c_{\mathrm{L}} + c_{\text{NL}} + c_{\mathrm{T}}}.   $$

In addition, the tumor-to-liver activity uptake ratio *T*/*N* can be estimated from the MAA SPECT/CT image

7 $$ T/N = \left(A_{\mathrm{T}} / m_{\mathrm{T}}\right) / \left(A_{\text{NL}} / m_{\text{NL}}\right).   $$

With Eq. 4, the lung shunt factor *L* and the tumor-to-liver activity uptake ratio *T*/*N*, the absorbed dose for lung, normal liver, and tumor partitions can be expressed as follows

8 $$ D_{\mathrm{L}} = \frac{49.38 \cdot A_{0} }{m_{\mathrm{L}}} L   $$

9 $$ D_{\text{NL}} = \frac{49.38 \cdot A_{0} }{ m_{\text{NL}} + T/N \cdot m_{\mathrm{T}} } \left(1-L\right)   $$

10 $$ D_{\mathrm{T}} = \frac{49.38 \cdot A_{0} }{ \frac{1}{T/N} \left(m_{\text{NL}} + T/N \cdot m_{\mathrm{T}} \right) } \left(1-L\right) = T/N \cdot D_{\text{NL}}.   $$

#### Extended partition model based on arterial territories

The classical MIRD partition model gathers large volumes of interest in one partition and assumes a uniform distribution of activity throughout each partition. Our territorial model keeps this basic assumption but introduces more partitions to the normal liver based on arterial liver territories, see the “Determination of arterial liver territories” section. Additionally, we permit several tumor partitions for non-connected tumors or metastases. The lung partition will be handled as in the PM, where lung dose is determined from the lung shunt fraction.

To set up the TM, we generalize the partition model for a set of partitions *Σ* in a first step. Therefore, we rewrite the normal liver and tumor dose of the partition model in Eqs. 9 and 10 by inserting Eq. 7 for the tumor-to-liver activity uptake ratio. Afterwards, we reformulate the absorbed dose yielding a similar expression compared to Eq. 4. The remaining factor is summarized in the fractional uptake of the liver *f*_NL_. With that the normal liver absorbed dose can be expressed by

11 $$\begin{array}{*{20}l} D_{\text{NL}} & = \frac{49.38 \cdot A_{0} \left(1-L\right) }{ m_{\text{NL}} + \frac{A_{\mathrm{T}}}{m_{\mathrm{T}}} \frac{m_{\text{NL}}}{A_{\text{NL}}} \cdot m_{\mathrm{T}} } \end{array} $$

12 $$\begin{array}{*{20}l} & = \frac{49.38 \cdot A_{0} \left(1-L\right) }{ m_{\text{NL} \left(1+ \frac{A_{\mathrm{T}}}{A_{\text{NL}}} \right)} } \end{array} $$

13 $$\begin{array}{*{20}l} & = \frac{49.38 \cdot A_{0} \left(1-L\right) }{m_{\text{NL}} } \cdot \frac{m_{\text{NL}} }{ m_{\text{NL} \left(1+ \frac{A_{\mathrm{T}}}{A_{\text{NL}}} \right)} } \end{array} $$

14 $$\begin{array}{*{20}l} & = \frac{49.38 \cdot A_{0} \left(1-L\right) }{m_{\text{NL}} } \cdot \mathrm{f}_{\text{NL}}.  \end{array} $$

The absorbed tumor dose can be expressed in a similar way by

15 $$\begin{array}{*{20}l} D_{\mathrm{T}} & = \frac{49.38 \cdot A_{0} \left(1-L\right) }{ m_{\mathrm{T}} \left(1+ \frac{A_{\text{NL}} }{A_{\mathrm{T}} }\right) } \end{array} $$

16 $$\begin{array}{*{20}l} & = \frac{49.38 \cdot A_{0} \left(1-L\right) }{ m_{\mathrm{T}}} \cdot \frac{m_{\mathrm{T}}}{ m_{\mathrm{T}} \left(1+ \frac{A_{\text{NL}} }{A_{\mathrm{T}} }\right) } \end{array} $$

17 $$\begin{array}{*{20}l} & = \frac{49.38 \cdot A_{0} \left(1-L\right) }{ m_{\mathrm{T}}} \cdot \mathrm{f}_{\mathrm{T}}.  \end{array} $$

The generalized absorbed dose for an arbitrary partition *i*∈*Σ* can be derived from Eqs. 14 and 17

18 $$\begin{array}{*{20}l} D_{i} & = \frac{49.38 \cdot A_{0} \left(1-L\right) }{ m_{i}} \cdot \frac{m_{i}}{ m_{i} \left(1+ \frac{A_{\Sigma\setminus i} }{A_{i} }\right) } \end{array} $$

19 $$\begin{array}{*{20}l} & = \frac{49.38 \cdot A_{0} \left(1-L\right) }{ m_{i}} \cdot \mathrm{f}_{i},  \end{array} $$

where *A*_*i*_ is the activity in partition *i* and *A*_*Σ*∖*i*_ describes the activity in *Σ* without partition *i*.

### SIRT activity calculation

#### Constrained partition activity calculation

The classical approach for activity calculation according to the original partition model is to solve the normal liver absorbed dose in Eq. 9 for the activity to deliver by setting a fixed limit for the mean dose to normal liver and lung [3]. Those limiting values are given in literature, e.g., dose thresholds given in the package insert for SIR-Spheres^®;^ [8] or thresholds discussed and published in [9]. Table 1 gives an overview of reported dose thresholds.

**Table 1:** Dose thresholds for lung, normal liver, and tumor according to [8] represented by *T*_1_ and dose thresholds *T*_2_ published and discussed in [9]

	*T*_1_	*T*_2_
*D*_L_	25 Gy	25 Gy
*D*_NL_	70 Gy	40 Gy
*D*_T_	> 100 Gy	> 100 Gy

This approach was directly transferred to the TM. To determine the activity to deliver, the mean normal liver dose over all liver partitions and the lung dose are restricted to thresholds given in Table 1.

#### BSA method

Alternatively, the activity to deliver can be determined by the body-surface-area (BSA) method [3] from the patient’s body height and weight

20 $$ \text{BSA}\left[\mathrm{m}^{2}\right] = 0.20247\times \text{height}\left[\mathrm{m}\right]^{0.725}\times \text{weight}\left[\text{kg}\right]^{0.425}   $$

21 $$ A\left[\text{GBq}\right] = \text{BSA} - 0.2 + \frac{V_{T}}{V_{\text{total}}},   $$

with tumor volume *V*_*T*_ and total liver and tumor volume *V*_total_.

This empirical method assumes a relation between the tumor size in the liver and the patient’s size. Despite reasonable concerns on the use of BSA, e.g., in [9], it is also given here for comparison purposes as it is still used in clinical practice.

### Patients, imaging, and image analysis

Data from five patients, who underwent SIRT at Klinikum Dresden-Friedrichstadt, Dresden, Germany, were retrospectively analyzed with regard to the proposed optimized liver territory-based partition model for activity and dose calculation. All patients received Tc-99m MAA during the evaluation procedure and Y-90 resin microspheres during the interventional treatment session. MAA SPECT/CT imaging was performed on a SymbiaT (Siemens Healthcare) machine. CT images were reconstructed using the B60s reconstruction kernel, one of the standard higher resolution body kernels [10]. SPECT images were acquired on a 128 × 128 × 81 matrix. It was ensured that the SPECT image includes the whole lung and liver. SPECT voxel spacing is 4.795 mm for all dimensions and CT voxel spacing is 0.9766 × 0.9766 × 5 mm in all cases. SPECT scan parameters were: number of angles 64, orbit range 180°, energy window 129–149 keV, collimator type parallel, reconstruction 3D ordered-subsets expectation maximization algorithm using 8 iterations, 4 subsets, and Gaussian filter with 6 mm FWHM. Pretreatment contrast-enhanced T1-weighted MR images were performed routinely on a GE Signa HDxt 1.5T MRI system (GE Healthcare). Related voxel sizes are given in Table 2. The MR images were used for liver and tumor segmentation, the arterial phase of a routine two-phase contrast-enhanced liver CT was used for artery segmentation. These CTs were acquired on a GE LightSpeed VCT (GE Healthcare). Scan parameters were: collimation 0.625 mm, pitch 0.984, rotation time 0.5 s, voltage 80 kV, current 320–680 mA. The segmentation was performed by an experienced radiological technician. The segmentation results are provided in SPECT/CT image domain via coregistration.

**Table 2:** Voxel sizes of acquired contrast-enhanced T1-weighted MR images

Patient	Voxel size
Pat1	1.562 × 1.562 × 2 mm
Pat2	0.7422 × 0.7422 × 5 mm
Pat3	0.7813 × 0.7813 × 2.2 mm
Pat4	0.7422 × 0.7422 × 2.2 mm
Pat5	0.7422 × 0.7422 × 2.4 mm

Volume and mass measurements were performed on the CT image using segmentation masks provided via coregistration. Lung segmentation was performed automatically on the CT images [11]. The liver territories of Pat1, Pat2, and Pat4 correspond to the eight Couinaud segments. For Pat5, the fourth segment was split into two separate segments. Pat3 has only four segments due to right hemihepatectomy. Segments five to eight were resected. Activity counts were determined on SPECT images.

## Results

Table 3 summarizes activity and dose calculation based on the PM and TM using thresholds *T*_1_, defined in Table 1. Further information on number of tumors, largest tumor size, and number of liver territories as well as activity values determined by the BSA method are given. For activity calculation, the main limiting factor in PM and TM is the maximum normal liver dose, except for Pat2, where the maximum dose to the lung limits the activity to deliver. In case of Pat2, the determined activity is equal for both methods, resulting in a similar lung and tumor dose. However, the normal liver dose is smaller in case of TM. For all other cases, normal liver dose reaches the maximum value defined by the threshold in both methods. Whereas lung dose is moderately larger in TM, tumor dose is significantly larger in TM than in PM. TM reaches the desired tumor dose > 100 Gy in all cases, whereas PM does not meet this requirement for Pat4. The BSA method provides the smallest activity values compared to PM and TM for all cases. For activity calculation using thresholds *T*_2_, defined in Table 1, the determined activity is for the PM in all five patients smaller than for the TM, see Table 4. In all cases, TM determines larger activity values to deliver than PM. The main limiting factor is mean liver dose except for Pat2 in TM, where mean lung dose limits the activity to deliver. In the cases of Pat1 and Pat2, the desired mean tumor dose > 100 Gy is achieved with both methods, in Pat3 only with TM, whereas it was not achieved in Pat3 with PM and in Pat4, and Pat5 with both methods.

**Table 3:** Activity and dose calculation were performed for five patients according to TM and the classical PM

		Pat1	Pat2	Pat3	Pat4	Pat5
Information	No. tumors	1	1	5	1	2
	Largest tumor [ml]	274.5	582.8	54.8	454.8	13.09
	No. liver territories	8	8	4	8	9
BSA model	*A*_0_ [GBq]	1.64	1.92	2.01	1.85	1.67
PM	*A*_0_ [GBq]	3.97	3.88	4.05	2.71	3.03
	*D*_L_ [Gy]	10.68	25.00	8.68	9.23	9.65
	*D*_NL_ [Gy]	70.00	43.41	70.00	70.00	70.00
	*D*_T_ [Gy]	287.95	190.55	167.48	88.75	144.69
TM	*A*_0_ [GBq]	5.97	3.88	4.78	4.62	3.31
	*D*_L_ [Gy]	16.12	24.78	10.62	15.73	10.75
	*D*_NL_ [Gy]	69.88	29.67	70.08	70.43	70.06
	*D*_T_ [Gy]	433.75	190.23	199.75	150.98	158.82

Thresholds *T*_1_ (see Table 1) were applied for normal liver and lung in activity calculation

**Table 4:** Activity and dose calculation were performed for five patients according to TM and the classical PM

		Pat1	Pat2	Pat3	Pat4	Pat5
PM	*A*_0_ [GBq]	2.27	3.58	2.31	1.55	1.73
	*D*_L_ [Gy]	6.10	23.03	4.96	5.27	5.52
	*D*_NL_ [Gy]	40.00	40.00	40.00	40.00	40.00
	*D*_T_ [Gy]	164.54	175.56	95.7	50.71	82.68
TM	*A*_0_ [GBq]	3.41	3.88	2.73	2.64	1.9
	*D*_L_ [Gy]	9.35	24.78	6.04	8.96	6.29
	*D*_NL_ [Gy]	39.90	29.67	39.94	40.25	40.00
	*D*_T_ [Gy]	247.71	190.23	113.96	86.13	90.88

For activity calculation, thresholds *T*_2_ (see Table 1) were applied for normal liver and lung

## Discussion

**Fig. 2:**
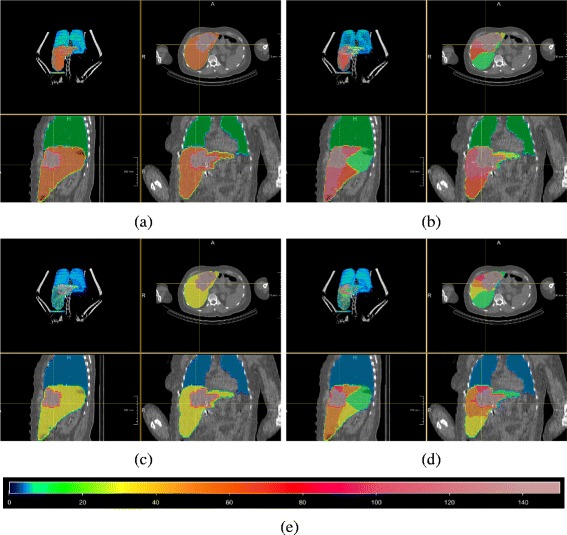
Visualization of dose distribution for Pat1 using PM (left) and TM (right) and normal liver thresholds *T*_1_ in the first row and *T*_2_ in the second row. The dose color bar, relating color to dose values, is given below. Each quartet shows axial, sagittal, coronal, and anterior 3D view. **a** PM activity and dose calculation using T_1_. **b** TM activity and dose calculation using T_1_. **c** PM activity and dose calculation using T_2_. **d** TM activity and dose calculation using T_2_. **e** Dose color bar in Gy

The partition model assumes a uniform distribution of activity throughout the mass of interest. The territorial model relies on the same assumption but uses more and therefore smaller partitions. Large tumor volumes and tumors with hypo- and hypervascular compartments will remain critical. Arterial liver territories might especially better approximate liver regions with uniform activity distribution due to the common dependence from one specific liver artery where Y-90 microspheres are delivered. Studies have shown that tumor-to-non-tumor activity uptake is not uniformly distributed [3]. Therefore, the partition model considers the fractional uptake factor for normal liver and tumor partition. Because the territorial model performs more locally, the activity uptake is also determined more locally. This highly local activity partitioning results in more local dose maps as demonstrated in Fig. 2. Therefore, the variability of dose to normal liver within the organ can be characterized more specific than in the classical partition model.

The calculated activity to deliver obviously depends on the threshold’s limiting dose to normal liver. However, the results also show a strong dependence on the method used for activity calculation. Typically, mean normal liver dose is the limiting factor in activity calculation, except for cases like Pat2, where lung dose limits the activity to deliver due to high lung shunting. Therefore, all determined activities are smaller when using the small normal liver threshold of *T*_2_ than using the threshold in *T*_1_. For *T*_2_, this results in insufficient tumor coverage for Pat3, Pat4, and Pat5 in case of PM. However, TM achieves the desired tumor dose for Pat3, and both methods were successful in Pat1 and Pat2. High tumor doses like 434 Gy for Pat1 with TM indicate a high tumor coverage in this case and suggest to extend the activity calculation by an additional constraint on the mean tumor dose. This will result in a smaller lung and normal liver dose exposure. A validation with post-interventional data should analyze the actually achieved doses with PM and TM and investigate whether locally higher liver doses can be accepted in return for sparing another major part of the liver.

The assumption that high normal liver counts in close neighborhood of tumor regions might originate from the activity at the tumor is demonstrated in Pat2. With *T*_1_ the activity to deliver is limited by the maximum lung dose using PM as well as TM. Therefore, both methods determine the same activity to deliver of 3.88 GBq. For PM, this results in a normal liver dose of 43.41 Gy. The TM excludes activity counts in a neighborhood of maximum Y-90 emitting range of 11 mm for normal liver dose calculation. This results in a significant lower normal liver dose of 29.67 Gy. The relatively small volume in close neighborhood to the tumor seems to have a large impact on normal liver dose. This might be caused by the beta particle range, producing a spill-out effect on the tumor, or partial volume effects. Because the specified margin of 11 mm is only approximately twice the original SPECT image resolution, see the “Patients, imaging, and image analysis” section, the assumption of excluding this region from normal liver dose calculation seems to be reasonable. All other patients show a similar behavior with normal liver dose limited by the threshold but a higher prescribed activity and therefore slightly higher lung dose and significant higher tumor doses. For Pat1 and Pat4, tumor doses could be increased by a factor of approximately 1.5, whereas the increase in tumor doses is smaller for Pat3 and Pat5. This observation suggests that higher tumor doses in TM are associated with a higher tumor volume. A clear correlation between the number of tumors and an activity increase from PM to TM was not demonstrated in the results of five patients. Further studies on larger databases should investigate this. A systematic evaluation and a detailed investigation of different margins have to be analyzed in a next step. Potential critical side effects have to be investigated carefully.

One advantage of the presented model is that it can be used in cases with several tumors present in liver tissue. The restriction of the PM to clearly differentiable tumors still remains true due to the dependence from segmentations. Therefore, tumors have to be differentiable and delineated on CT images or coregistered MR images rather than on SPECT images. The margin around the tumor was determined here by the maximum Y-90 emitting range of 11 mm. Other possibilities, e.g., margin selection depending on SPECT image resolution or mean Y-90 emitting range, might be considered and its influence should be analyzed. The impact of image noise as well as segmentation and coregistration accuracy should be investigated.

The presented approach, as well as the partition model in general, assumes that MAA is a suitable surrogate for Y-90 microspheres and that the MAA particle distribution is similar to the Y-90 particle distribution. This is controversially discussed: A high correlation of MAA and calibrated beta-probe was shown in [12], and [3] confirms that the assumption of microsphere and MAA particle distribution similarity introduces less error into dose calculations than the assumption of uniform activity throughout a volume of interest. The recommendation of Dezarn et al. [3] to use PM, which is relying on the MAA SPECT/CT, is contradicted by results of Wondergem et al. [13]. Poor correlation between MAA and Y-90 is also reported in [14] caused by systematic errors, like differences in catheter position, injection techniques, or differences in particle sizes, flow hemodynamics, or disease progression. A good correlation of predictive MAA SPECT-based dosimetry with post-radioembolization Y-90 PET dosimetry was demonstrated in [15]. Also, [16] showed a good correlation of MAA SPECT and Y-90 PET tumor-to-normal uptake ratios. Gnesin et al. [17] showed that the MAA SPECT provides a good estimate of absorbed doses compared to post-treatment PET for tumor and non-tumor tissues in HCC radioembolization. Despite this, MAA SPECT imaging is currently the only possibility for predictive three-dimensional dose assessment.

The reliance upon MAA SPECT is one of the main limitations for a partition-based approach for SIRT activity and dose calculation and also applies to the presented approach. A comparison of predictive TM dosimetry based on MAA SPECT and post-interventional TM dosimetry based on Y-90 PET/CT imaging is planed to be investigated in a next step. As a prospect for the future, the proposed liver territory-based partition model is designed to enable SPECT-independent activity calculation and dose prediction under the condition of an artery-based simulation for particle distribution. MAA SPECT imaging would be used then only to respect safety-oriented tasks, e.g., estimation of extra-hepatic shunting.

Recently, more advanced methods for voxel-based dose calculation, such as Monte Carlo [18], dose point kernel [19], and local deposition methods [20], have been developed. However, they still rely on the MAA SPECT/CT and the recommendation of the microsphere manufacturers [8] remains on activity and dose calculation based on the PM which is widely used in clinical practice. Reason for this is, it can be easily performed and offers a practical option for individual activity planning [21]. Provided that there is a reliable artery-based simulation for particle distribution, the proposed liver territory-based partition model enables SPECT-independent activity calculation and dose prediction based on the territories. This is the decisive advantage over three-dimensional voxel-based dosimetry methods, which cannot achieve that.

## Conclusions

An extended partition model based on arterial liver territories is proposed for SIRT activity and dose calculation. This method is able to better account for non-uniformly distributed activity in normal liver tissue. By this, the proposed method is also able to provide a more local dose distribution. Compared to the classical partition model, both methods predict the same normal liver dose, whereas the predicted activity and dose for lung and tumor tissue is lower in the classical model than in the territorial model using a 11-mm margin. This leads to the conclusion that tumors increase normal liver dose and that excluding a defined, small region around tumors from normal liver in case of normal liver dose calculation based on a partition model can estimated liver partition dose more precise. Studies on a larger database and post-interventional images should investigate this further.
